# Impact of co-infection with *Plasmodium berghei* ANKA in *Leishmania major*-parasitized mice on immune modulation and cutaneous leishmaniasis

**DOI:** 10.1371/journal.pntd.0013302

**Published:** 2025-07-28

**Authors:** Uyla Ornellas-Garcia, Lucas Freire-Antunes, Marcos Rangel-Ferreira, Carina Heusner Gonçalves de Sousa, Mônica Lucas Ribeiro-Almeida, Cláudio Tadeu Daniel-Ribeiro, Patricia Cuervo, Flávia Lima Ribeiro-Gomes

**Affiliations:** 1 Laboratório de Pesquisa em Malária, Instituto Oswaldo Cruz, Fundação Oswaldo Cruz, Rio de Janeiro, Brazil; 2 Centro de Pesquisa, Diagnóstico e Treinamento em Malária, Fundação Oswaldo Cruz, Rio de Janeiro, Brazil; 3 Laboratório de Pesquisa em Leishmanioses, Instituto Oswaldo Cruz, Fundação Oswaldo Cruz, Rio de Janeiro, Brazil; Centro de Pesquisa Gonçalo Moniz-FIOCRUZ/BA, BRAZIL

## Abstract

**Background:**

Malaria and leishmaniasis are vector-borne diseases responsible for a significant number of deaths worldwide. Despite the co-endemicity of these diseases in regions with tropical and subtropical climates, our understanding of the complex interplay between *Plasmodium* spp. and *Leishmania* spp. co-infections on host immune response and resultant disease outcomes remains limited.

**Methodology/Principal findings:**

This study employs C57BL/6 mice co-infected with *Leishmania major* and *Plasmodium berghei* ANKA, well-established models of cutaneous leishmaniasis and experimental cerebral malaria, respectively. Our findings demonstrate that an acute infection with *P. berghei* ANKA mitigates the progression of ongoing cutaneous leishmaniasis, as evidenced by a reduction in lesion size and parasite burden in the dermis of *L. major*-infected mice. Co-infection also led to elevated serum levels of TNF compared to the levels observed in mice infected with *L. major* alone, which may contribute to a more effective control of the *Leishmania* parasite. Furthermore, co-infected mice exhibited reduced recruitment of activated T cells and inflammatory monocytes to the site of *L. major* infection. As inflammatory monocytes can be exploited by *Leishmania* as host cells that support parasite replication, their reduced infiltration may limit parasite growth. This diminished cellular infiltration is likely to contribute to reduced local inflammation, thereby limiting tissue damage and resulting in smaller lesion size.

**Conclusions/Significance:**

These findings elucidate the potential cross-regulation of immune responses in co-infections, underscoring the necessity to consider co-infecting pathogens in disease management and therapeutic strategies in endemic areas.

## Author summary

The co-endemicity of malaria and leishmaniasis affects thousands of individuals annually, potentially complicating the diagnosis and resolution of both diseases. However, few studies have explored how the presence of a secondary infection might modulate the host immune response, either exacerbating or suppressing it. In this study, we utilize experimental models to investigate the impact of *Plasmodium berghei* ANKA infection on the progression of *Leishmania major* infection, the causative agent of cutaneous leishmaniasis in both humans and mice. Our findings demonstrate that, while the clinical course of malaria remains unaffected by co-infection, the progression of cutaneous leishmaniasis is significantly altered. Co-infection leads to reduced tissue damage and a lower *Leishmania* parasite load in the dermis. This phenomenon can be attributed to a decrease in the recruitment of inflammatory monocytes and activated T cells to the site of infection, leading to reduced inflammation and lower availability of host cells, particularly inflammatory monocytes, that support *Leishmania* replication.

## Introduction

Vector-borne diseases, including malaria and leishmaniasis, are endemic in tropical and subtropical regions, impacting a substantial portion of the global population and responsible for a significant number of deaths annually [1]. The World Malaria Report (2024) indicates that 263 million cases of malaria caused by *Plasmodium* spp. were reported in 2023, resulting in 597,000 deaths [2]. The mortality rate associated with malaria is closely linked to the development of severe disease manifestations, such as cerebral malaria (CM) [3]. Although the immunopathological mechanisms are not fully understood, CM is intrinsically related to parasitized red blood cells (pRBCs) adhering to the endothelium of cerebral microvasculature. Furthermore, an exacerbated type 1 immune response is observed, which triggers heightened endothelial activation, amplified pRBCs adherence, mechanical obstruction of cerebral blood vessels, decreased blood flow and oxygen delivery, and ultimately, tissue damage and blood-brain barrier disruption [4,5].

Similarly, *Leishmania* parasites cause a spectrum of diseases responsible for nearly 1 million new cases annually [2], and more than 4000 deaths were reported in the last decade [6]. The pathophysiology of leishmaniasis is determined by a number of interacting factors, including parasite species, host-related characteristics (particularly immune responses), and the occurrence of co-infections [7]. The disease can range from self-limiting skin lesions to fatal visceral forms if left untreated [7]. Cutaneous leishmaniasis (CL) represents the most prevalent form of the disease. Despite the World Health Organization (WHO) reporting 205,990 cases in 2022, the actual number of cases is estimated to exceed 600,000, due to underreporting of the disease [8]. In CL caused by *L. major* and *L. braziliensis*, an inflammatory type-1 immune response is typically associated with parasite control [9,10]; however, it is not always correlated with less severe pathology [11].

Leishmaniasis and malaria are co-endemic in several countries [12–14], and co-infection between these two pathogens is a recurrent phenomenon [14–22]. Nevertheless, the study of cases of co-infection and their clinical and immune implications for the host is an overlooked issue.

To address this knowledge gap, the present study utilized well-established models in C57BL/6 mice. These mice are known for their inherent resistance to *L. major* (Friedlin strain) infection, developing self-healing lesions following intradermal inoculation. This resistance is associated with a robust Th1-polarized immune response, characterized by IFN-γ and TNF production and macrophage activation, which are essential for parasite elimination [23,24]. Conversely, C57BL/6 mice demonstrate a marked susceptibility to *P. berghei* ANKA infection, thereby serving as the principal model for experimental cerebral malaria (eCM). As indicated by the extant literature, this infection has been shown to lead to neurological disorders and a high mortality rate between six and nine days post-infection [5,25]. The pathogenesis in this model mirrors human CM, with excessive production of IFN-γ and TNF contributing to immune-mediated vascular damage [4,26]. Therefore, to explore the immunological interplay between these two protozoan parasites and their impact on disease outcomes, C57BL/6 mice were first infected with *L. major* and subsequently challenged with *P. berghei* ANKA.

Our findings demonstrate that the immune response induced by *P. berghei* ANKA modulates the ongoing response in C57BL/6 mice previously infected with *L. major,* leading to reduced recruitment of T cells and inflammatory monocytes to the dermal site of *Leishmania* infection, along with increased systemic levels of TNF. This immunomodulation alters the clinical course of CL, as evidenced by reduced lesion size and parasite load.

## Materials and methods

### Ethics statement

Female C57BL/6 mice aged 6–8 weeks were obtained from the Instituto de Ciências e Tecnologia em Biomodelos (ICTB), Fiocruz. The animals were maintained under controlled light and temperature conditions and provided with *ad libitum* access to food and water. All animal procedures were conducted in strict accordance with relevant regulations and guidelines. All experimental protocols were approved by the local Ethical Committee for Animal Use (Comissão de Ética no Uso de Animais, CEUA) of the Instituto Oswaldo Cruz, Fundação Oswaldo Cruz - FIOCRUZ (license numbers L030-2017 and L-030–2017 A1).

### Parasites and infection

*Leishmania major* Friedlin FV1 (MHOM/IL/80/FN) parasites were cultured at 26°C in Schneider medium supplemented with 100U/mL penicillin, 100μg/mL of streptomycin, 2% sterile human urine, and 10% heat-inactivated fetal bovine serum (FBS) (Gibco). These parasites were utilized up to the fourth *in vitro* passage. Metacyclic promastigotes of *L. major* were isolated through a 10% Ficoll gradient centrifugation (1690g, 10 minutes, 28ºC) from stationary *in vitro* cultures that had been maintained for 5–6 days [27]. Mice were infected with 2 x10^5^ metacyclic forms via injection into the ear dermis.

*P. berghei* ANKA expressing a green fluorescent protein was generated as previously described [28] and supplied by the Malaria Research and Reference Reagent Resource Center-MR4, Manassas, VA (MR4 number: MRA-865). The blood samples containing pRBCs were cryopreserved in liquid nitrogen. Before infecting the experimental groups, a 100 µL aliquot of the blood was thawed and inoculated intraperitoneally into healthy mice. Four to five days later, blood samples were obtained from the animals via cardiac puncture, and parasitemia and red blood cell (RBC) counts were estimated. Subsequently, the mice comprising the experimental group were intraperitoneally injected with 10^6^ fresh pRBCs.

The experimental design, detailing the sequence of infections, is described in S1 Fig. Mice were randomly assigned to four groups: one group infected with *L. major* only (*Lm* group), one infected with *P. berghei* ANKA only (*Pb*A group), one co-infected with both *L. major* and *P. berghei* ANKA (*Lm* + *Pb*A group), and one group of uninfected animals (Naïve group).

### Evaluation of *Plasmodium berghei* ANKA parasitemia and body temperature

On the sixth day following infection with *P. berghei* ANKA, parasitemia and body temperature were assessed. The parasitemia was determined by flow cytometry, based on the percentage of GFP^+^pRBCs. Rectal temperature was determined by using a thermocouple probe (Oakton Acorn; Oakton Instruments, IL, USA). Furthermore, the occurrence of clinical signs, including lethargy, ataxia, seizures, and coma, was monitored daily.

### Evaluation of *L. major* lesion size and parasite load

The thickness and diameter of dermal lesions resulting from *L. major* infection were evaluated over a period of approximately 16 weeks using a digital caliper (DIGIMESS). The parasite loads in the ear and the draining lymph node were determined through limiting dilution analysis [29,30]. Briefly, tissue suspensions were serially diluted in 96-well flat-bottom culture plates containing Schneider medium supplemented with 20% heat-inactivated FBS and 2% sterile human urine. The plates were examined microscopically for the presence of promastigotes on days 4, 7, and 14. The parasite burden in each tissue was calculated by counting the number of parasite-positive wells.

### Measurement of serum cytokine levels

The cytokine levels in the serum of the experimental groups were evaluated using a BD Cytometric Bead Array Kit for Mouse Th1/Th2/Th17 Cytokines (BD Biosciences, San Jose, CA) in accordance with the manufacturer’s instructions.

### Processing of tissue samples from the ear, draining lymph node, and spleen

The preparation of the ear tissue was conducted in accordance with the methodology previously described [31] with minor modifications. In brief, the ears were removed and promptly immersed in 70% ethanol for a period of 2–5 minutes, followed by air-drying. The two layers of ear dermis were separated and placed in RPMI medium containing 0.160 mg/ml Liberase CI purified enzyme blend (Roche Diagnostics Corp.), and incubated for 1 hour and 30 minutes at 37°C. Following digestion, the tissue was homogenized through a 70 μm-pore-size cell strainer using a syringe plunger in RPMI medium containing 0.05% DNase I (SIGMA, ALDRICH). The resulting cell suspension was then subjected to centrifugation (609g, 8 minutes, 4ºC) and resuspended in 1 mL of complete Schneider medium.

The ear-draining lymph node was excised and transferred to separate Eppendorf tubes containing 300 µL each of supplemented Schneider medium. The lymph node was macerated individually using a Pellet Pestle Motor (Kontes) until complete dissociation of the organ was achieved. Thereafter, the volume was adjusted to 700 µL using Schneider medium. The total number of cells was calculated after counting them using a Neubauer chamber.

The spleens were carefully harvested, cut into smaller pieces using scissors, and subsequently homogenized in cold phosphate-buffered saline (PBS) using a 70 μm-pore cell strainer to obtain a single cell suspension. The resulting cell suspensions were subjected to centrifugation (609g, 8 minutes, 4ºC), and the pellet was resuspended in Red Blood Cell Lysing Buffer (Sigma, Life Science) to eliminate RBCs. The splenocyte suspensions were then thoroughly washed and resuspended in PBS containing 5% FBS. The total count of splenocytes was determined by diluting the cell suspension in Trypan blue and counting them using a Neubauer chamber.

### Immunophenotyping of myeloid and lymphoid cell populations

Following the extraction and processing of spleen, draining lymph nodes, and ear tissues from both infected and uninfected animals, immunolabeling was performed. Specifically, 1 x10^6^ cells from the spleen and draining lymph nodes, as well as half of the homogenized ear tissue, were washed and labeled with various combinations of anti-mouse antibodies. To identify lymphoid populations, cells were incubated on ice for 20 minutes with a pool of antibodies, including anti-Fc-γ III/II (CD16/32) receptor antibody (2.4G2), anti-TCRβ PE (H57-597) or anti-CD3 PercpCy7 (145-2C11), anti-CD4 APC-H7 (GK1.5), anti-CD8 PeCy7 (53-6.7), and anti-CD62L FITC (MEL-14). To identify myeloid populations, cells were incubated with the following antibodies: anti-Fc-γ III/II (CD16/32) receptor antibody (2.4G2), anti-CD11b PeCy7 (M1/70), anti-Ly6C APC-Cy7 (AL-21), and anti-Ly6G APC (1A8). In some experiments, to assess IFN-γ production, cells were fixed, permeabilized, and incubated with anti-IFN-γ APC (XMG1.2) antibodies following surface staining. All antibodies utilized in this study were from BD Biosciences. Following the incubation period, the cells were washed twice prior to acquisition. Samples were acquired using either FACSCanto II (BD Biosciences) or CytoFLEX (Beckman Coulter) flow cytometer. Data were analyzed using FlowJo 10.0 software (BD Biosciences).

### Statistical analysis

All graphs and statistical analyses were made using GraphPad Prism version 8.0 (GraphPad Software, USA). Comparison between the two groups was conducted using the unpaired t-test with a 95% confidence level. To compare more than two groups, a One-way ANOVA test was conducted, followed by a Tukey’s multiple comparison test. Only results with p-values less than 0.05 (p < 0.05) were considered statistically significant.

## Results

### Acute infection with *Plasmodium berghei* ANKA in *Leishmania major*-parasitized mice reduces dermal lesion size and parasite load in those with cutaneous leishmaniasis

**Fig 1:**
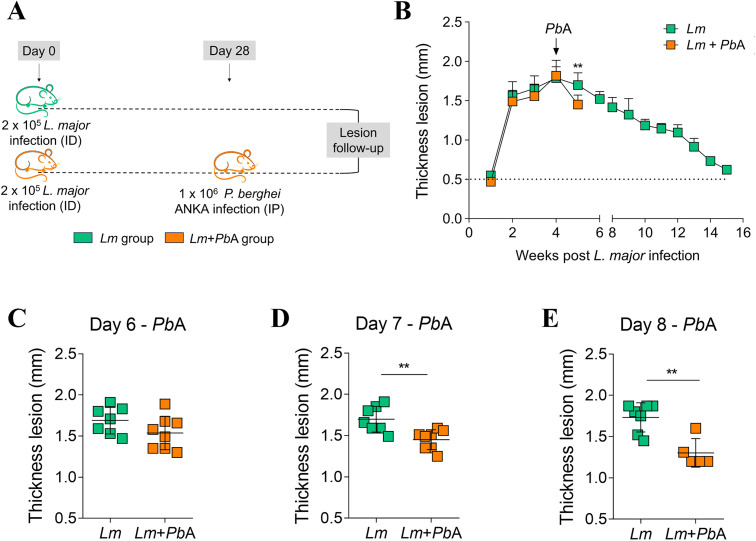
Co-infection with *P. berghei* ANKA on day 28 of *L. major* infection reduces dermal lesion in C57BL/6 mice with cutaneous leishmaniasis. On the 28th day following infection with *L. major*, mice were either infected or not with *P. berghei* ANKA (A). The development of dermal lesions was monitored and measured using a digital caliper on a weekly basis (B) prior to *P. berghei* ANKA infection, and on a daily basis, specifically on days 6 (C), 7 (D), and 8 (E) following infection with *P. berghei* ANKA. The experimental groups were designated as *Lm* (mice infected with *L. major* only) and *Lm* + *Pb*A (mice co-infected with both *L. major* and *P. berghei* ANKA). The data presented are representative of a single experiment, with n = 7-8 mice per group. Asterisks indicate statistically significant differences, analyzed by Student’s t test, considering p < 0.05. Mouse illustration obtained from Openclipart (https://openclipart.org), public domain (CC0 license).

**Fig 2:**
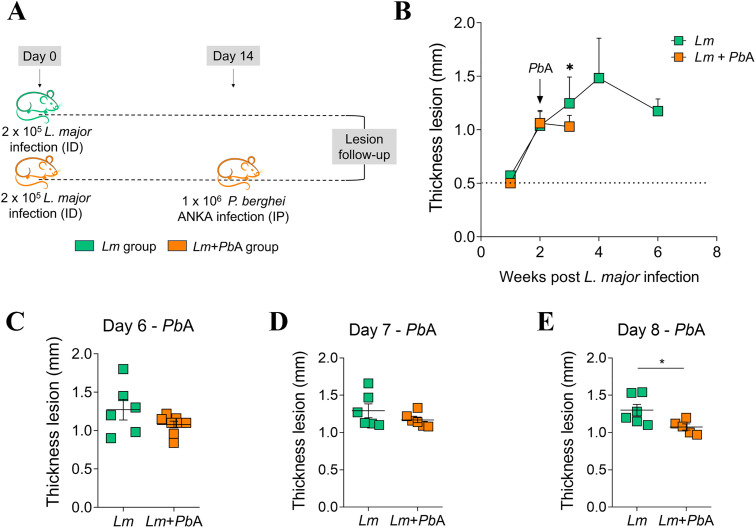
Co-infection with *P. berghei* ANKA on day 14 of *L. major* infection reduces dermal lesions in C57BL/6 mice with cutaneous leishmaniasis. On the 14th day following infection with *L. major*, mice were either infected or not with *P. berghei* ANKA (A). The development of dermal lesions was monitored and measured using a digital caliper on a weekly basis (B) prior to *P. berghei* ANKA infection, and on a daily basis, specifically on days 6 (C), 7 (D), and 8 (E) following infection with *P. berghei* ANKA. The experimental groups were designated as *Lm* (mice infected with *L. major* only) and *Lm* + *Pb*A (mice co-infected with both *L. major* and *P. berghei* ANKA). The data presented are representative of a single experiment, with n = 6-8 mice per group. Asterisks indicate statistically significant differences, analyzed by Student’s t test, considering p < 0.05. Mouse illustration obtained from Openclipart (https://openclipart.org), public domain (CC0 license).

To assess whether *Plasmodium* infection might impact the clinical course of an ongoing *Leishmania* infection, we used well-known experimental models of eCM [32–34] and CL [24]. *P. berghei* ANKA infection of C57BL/6 mice has been shown to induce eCM, resulting in death 6–9 days post-infection [32,33]. In contrast, *L. major* infection of C57BL/6 mice has been observed to result in a cutaneous lesion that self-resolves within 12 weeks after inoculation [23,35]. In this study, mice were injected into the ear dermis with metacyclic promastigote forms of *L. major*, and at the peak (day 28th) or beginning (day 14th) of the dermal lesion development*,* the animals were co-infected or not with *P. berghei* ANKA (Figs 1A and 2A). Co-infection with *P. berghei* ANKA reduced the cutaneous lesion size of mice previously parasitized with *L. major*, independently of the time (28- or 14-days post-*L. major* infection) (Figs 1B and 2B)*.* Lesion thickness between co-infected (*Lm* + *Pb*A group) and *L. major-*only infected mice (*Lm* group) was statistically different at days 7 (Fig 1D) and 8 post-*P. berghei* ANKA infection (Figs 1E and 2E) exhibiting a reduction in the *Lm* + *Pb*A group. Note that although the reduction in lesion thickness appeared statistically different earlier (on day 7 post-*P. berghei* ANKA infection) in the group co-infected at day 28, no such difference was observed in the group co-infected at day 14. However, any direct comparisons between the two timepoints should be interpreted with caution, as these experiments were conducted independently. Taken together, both datasets support the notion that *P. berghei* ANKA co-infection mitigates the progression of CL.

Given the similarity of the results obtained from our co-infection analyses with *P. berghei* ANKA at 28- and 14-days post-infection with *L. major* regarding the lesion progression, we opted to proceed with our analysis by maintaining the co-infection at day 14 post-*L. major* infection. This timepoint represents a critical phase of active lesion development, marked by intense parasite replication and immune cell recruitment, thereby providing a valuable window to investigate the immediate impact of a secondary infection. While this earlier stage may not fully capture the extent of *P. berghei* ANKA-mediated modulation, it presents a technically feasible and biologically informative opportunity to dissect the initial host-pathogen interactions during co-infection.

**Fig 3:**
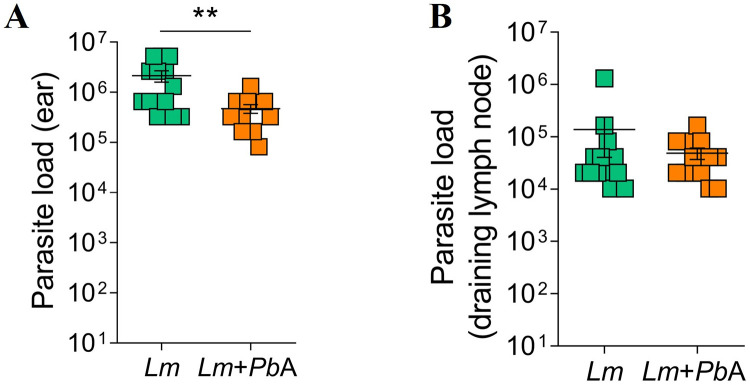
Co-infection with *P. berghei* ANKA reduces parasite load in the ear of *L. major*-infected C57BL/6 mice. On the 14th day following infection with *L. major*, mice were either infected or not with *P. berghei* ANKA. Parasite load was assessed on day 20 of *L. major* infection (corresponding to day 6 of *P. berghei* ANKA infection) in the ear lesion (A) and the draining lymph node (dLN) (B). The experimental groups were designated as *Lm* (mice infected with *L. major* only) and *Lm* + *Pb*A (mice co-infected with both *L. major* and *P. berghei* ANKA). The data represent a pool of two independent experiments, with n = 13 mice/group. Asterisks indicate statistically significant differences, analyzed by Student’s t test, considering p < 0.05.

The co-infection of *L*. *major*-parasitized mice at day 14 with *P. berghei* ANKA led to a reduction in the load of *Leishmania* parasite in the ear dermis when compared to *L. major*-only infected mice (Fig 3A). No difference was observed in the parasite load between *Lm* + *Pb*A and *Lm* groups in the skin-draining lymph nodes (Fig 3B).

**Fig 4:**
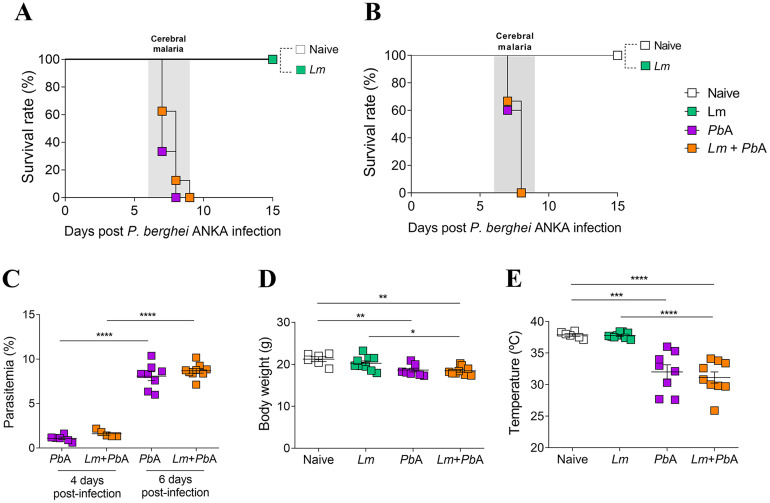
Malaria progression analysis shows no difference between co-infected and *P. berghei* ANKA-only infected C57BL/6 mice. On the 28th day (A) and 14th day (B) following infection with *L. major*, mice were infected with *P. berghei* ANKA and monitored daily for survival. Mice infected with *P. berghei* ANKA only or *L. major* only, as well as uninfected controls, were also monitored over time. (C) Parasitemia on days 4 and 6, and (D) body weight and (E) temperature on day 6 post-*P. berghei* ANKA infection in mice co-infected on day 14 of *L. major* infection. The experimental groups were designated as Naïve (uninfected animals), *Lm* (mice infected with *L. major* only), and *Lm* + *Pb*A (mice co-infected with both *L. major* and *P. berghei* ANKA). Asterisks indicate statistically significant differences, as analyzed by One-Way ANOVA with p < 0.05.

Of note, the *Lm* + *Pb*A and *Pb*A groups exhibit comparable survival rates, with mice from both groups succumbing to eCM between 6 and 9 days post-*P. berghei* ANKA infection, regardless of whether the co-infection was established on day 28 (Fig 4A) or day 14 (Fig 4B) following *L. major* infection. Furthermore, parasitemia, body weight, and temperature were also assessed to provide a more comprehensive characterization of the disease course. Parasitemia (Fig 4C) measured on days 4 and 6 post-*P. berghei* ANKA infection showed similar kinetics in both co-infected and *P. berghei* ANKA-only infected mice. Body weight (Fig 4D) and temperature (Fig 4E) measurements on day 6 post-*P. berghei* ANKA infection revealed similar patterns of weight loss and hypothermia (used to define late stage of eCM in various studies [36,37] in both the *Lm* + *Pb*A and *Pb*A groups, in contrast to the Naive and *Lm* groups. Together, these results indicate that a pre-existing *L. major* infection does not significantly alter the clinical progression of *P. berghei* ANKA infection in this co-infection model.

### Co-infection with *Plasmodium berghei* ANKA results in reduced T cell and myeloid cell counts at the dermal site of *Leishmania major* infection and in the skin-draining lymph node

**Fig 5:**
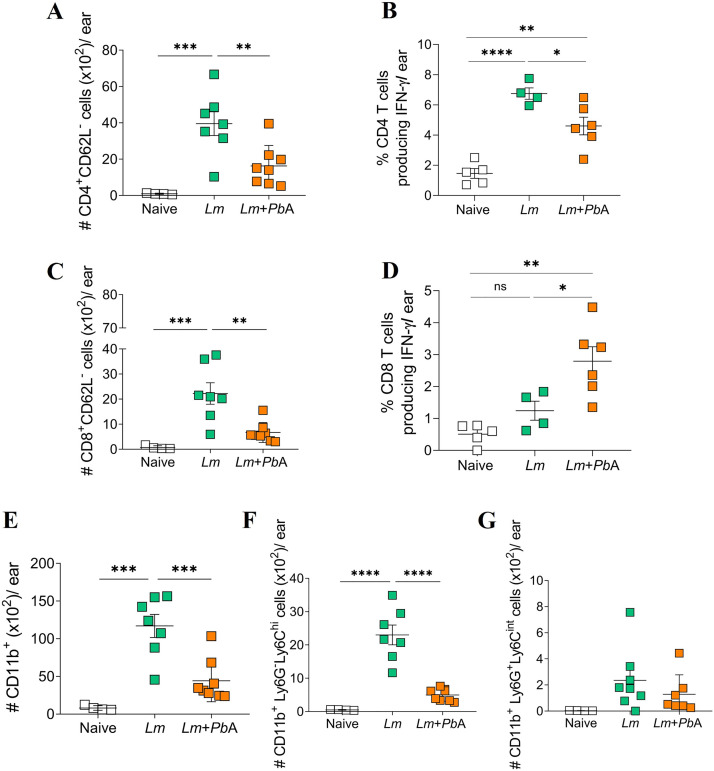
Recruitment of T cells and myeloid cells to the site of *L. major* infection is diminished during co-infection. Flow cytometry was employed to assess the total number of CD4 T cells (CD3^+^CD4^+^CD62L^-^) (A), CD8 T cells (CD3^+^CD8^+^CD62L^-^) (C), the percentage of IFN-γ^+^ CD4 T cells (B) and IFN-γ^+^ CD8 T cells (D), as well as the total number of myeloid cells (CD11b^+^) (E), inflammatory monocytes (CD11b^+^Ly6G^-^Ly6C^hi^) (F), and neutrophils (CD11b^+^Ly6G^+^Ly6C^int^) (G) recruited to the ear of the different experimental groups on day 20 of *L. major* infection (corresponding to day 6 of *P. berghei* ANKA infection). The experimental groups were designated as Naïve (uninfected animals), *Lm* (mice infected with *L. major* only), and *Lm* + *Pb*A (mice co-infected with both *L. major* and *P. berghei* ANKA). The data are representative of two independent experiments with n = 4-8 mice/group. Statistical analyses were performed by One-way ANOVA with Tukey’s multiple comparisons test. Values of p < 0.05 were considered statistically significant.

In order to determine the biological processes initiated by *P. berghei* ANKA infection that could be influencing *L. major* pathology, we evaluated the recruitment of CD4 and CD8 T cells and myeloid cell subpopulations to the site of *L. major* infection and the T cell numbers in the draining lymph nodes 6 days post-*P. berghei* ANKA infection. To this end, we established gate strategies for the selective identification of the cells of interest in both tissues (S2 and S3 Figs). Flow cytometry analysis of the dermal tissue revealed a robust recruitment of CD4 (CD3^+^CD4^+^CD62L^-^) and CD8 (CD3^+^CD8^+^CD62L^-^) T cells in the ear dermis of *L. major*-only infected mice, as compared to the Naïve group (Fig 5A and 5C). However, acute infection with *P. berghei* ANKA in *L. major*-parasitized mice resulted in decreased number of these cells in the ear dermis (Fig 5A and 5C). Among the CD4 T cells in the ear dermis, it was possible to identify an increase in the percentage of IFN-γ^+^ cells in *L. major*-only infected mice, which was reduced following co-infection with *P. berghei* ANKA (Fig 5B). In contrast, co-infection resulted in an increase in the percentage of IFN-γ^+^ CD8 T cells (Fig 5D). However, it is noteworthy that the total number of CD8 T cells was significantly reduced in co-infected mice (Fig 5C). Moreover, the absolute number of IFN-γ^+^ CD8 T cells did not differ between co-infected mice and those infected with *L. major* alone (S5 Fig), suggesting that the observed increase in the percentage of IFN-γ^+^ CD8 T cells does not correspond to a biologically significant change in cell numbers. As anticipated, all CD4 and CD8 T cells present at the site of infection exhibited the CD62L^-^ phenotype, indicative of activation.

The recruitment of myeloid cells (CD11b^+^) was also evaluated across all experimental groups, including inflammatory monocytes (CD11b^+^Ly6G^-^Ly6C^hi^) and neutrophils (CD11b^+^Ly6G^+^Ly6C^int^). Notably, *L. major* infection induced a significant increase in the total number of CD11b^+^ cells and inflammatory monocytes. However, in *L. major*-parasitized mice co-infected with *P. berghei* ANKA, the overall count of myeloid cells and inflammatory monocytes at the site of *L. major* infection was reduced compared to animals infected with *L. major* only (Fig 5E and 5F). No difference was observed in the total number of neutrophils between the groups in the dermis (Fig 5G).

**Fig 6:**
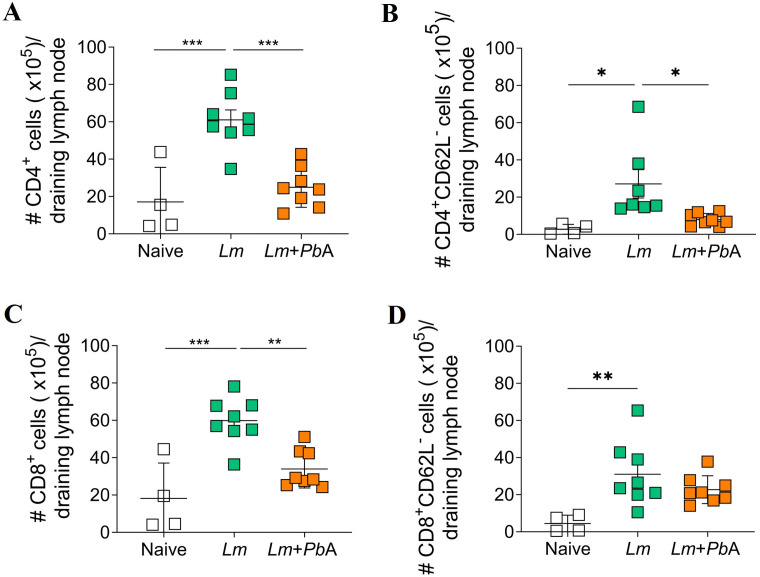
Co-infection with *P. berghei* ANKA reduces the number of T cells in the draining lymph node associated with the *L. major* infection site. Flow cytometry was employed to assess the total number of CD4 T cells (CD3^+^CD4^+^) (A), activated CD4 T cells (CD3^+^CD4^+^CD62L^-^) (B), CD8 T cells (CD3^+^CD8^+^) (C), and activated CD8 T cells (CD3^+^CD8^+^CD62L^-^) (D) present in the draining lymph node of the experimental groups on day 20 of *L. major* infection (corresponding to day 6 of *P. berghei* ANKA infection). The experimental groups were designated as Naïve (uninfected animals), *Lm* (mice infected with *L. major* only), and *Lm* + *Pb*A (mice co-infected with both *L. major* and *P. berghei* ANKA). The data are representative of two independent experiments with n = 4-8 mice/group. Statistical analyses were performed by One-way ANOVA with Tukey’s multiple comparisons test. Values of p < 0.05 were considered statistically significant.

A comparable phenomenon was observed in the draining lymph node. The total number of CD4 T cells (CD3^+^CD4^+^), activated CD4 T cells (CD3^+^CD4^+^CD62L^-^), CD8 (CD3^+^CD8^+^) and activated CD8 T cells (CD3^+^CD8^+^CD62L^-^) significantly increased following *L. major* infection, but declined in co-infected animals, except for activated CD8 T cells, which remained unchanged compared to animals infected only with *L. major* (Fig 6A–6D).

In summary, our analysis of immune cell populations in different tissues from *L. major*-only infected mice and co-infected mice underscored that the introduction of *P. berghei* ANKA co-infection during ongoing CL results in a reduction of T cells at both the site of *L. major* infection and the draining lymph node. Furthermore, there was a marked decrease in inflammatory monocytes at the site of infection.

### Co-infection of *Leishmania major-*parasitized mice with *Plasmodium berghei* ANKA induces alterations in the cell counts recovered from the spleen

**Fig 7:**
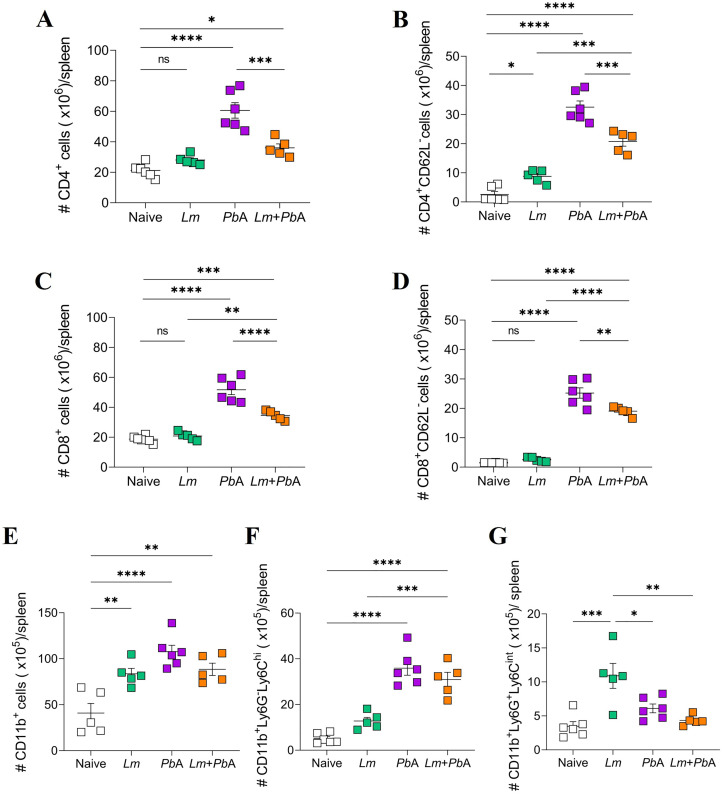
Analysis of T cell and myeloid cell populations in the spleen during *L. major* and *P. berghei* ANKA infections. Flow cytometry was employed to assess the total number of CD4 T cells (CD3^+^CD4^+^) (A), activated CD4 T cells (CD3^+^CD4^+^CD62L^-^) (B), CD8 T cells (CD3^+^CD8^+^) (C), activated CD8 T cells (CD3^+^CD8^+^CD62L^-^) (D), myeloid cells (CD11b^+^) (E), inflammatory monocytes (CD11b^+^Ly6G^-^Ly6C^hi^) (F) and neutrophils (CD11b^+^Ly6G^+^Ly6C^int^) (G) present in the spleen of the experimental groups on day 20 of *L. major* infection (corresponding to day 6 of *P. berghei* ANKA infection). The experimental groups were designated as Naïve (uninfected animals), *Lm* (mice infected with *L. major* only), *Pb*A (mice infected with *P. berghei* ANKA only), and *Lm* + *Pb*A (mice co-infected with both *L. major* and *P. berghei* ANKA). The data are representative of two independent experiments with n = 5-6 mice/group. Statistical analyses were performed by One-way ANOVA with Tukey’s multiple comparisons test. Values of p < 0.05 were considered statistically significant.

We next evaluated the number of T cells and myeloid cells in the spleen, a pivotal organ in the immune response during the erythrocytic phase of *Plasmodium* spp. infection [38]. Using flow cytometry and gating strategies to delineate the cells of interest (S4 Fig), an increase in the numbers of total (Fig 7A and 7C) and activated (Fig 7B and 7D) CD4 and CD8 T cells in the spleens of mice infected with *P. berghei* ANKA alone or co-infected (*Pb*A and *Lm* + *Pb*A groups) was observed in comparison to uninfected animals (Fig 7A–7D). Interestingly, the number of CD4 (activated) and CD8 (total and activated) T cells in co-infected mice consistently exceeded the values observed in mice infected only with *L. major*, although their numbers remained lower than in mice infected exclusively with *P. berghei* ANKA (Fig 7A–7D).

Turning our attention to myeloid cell subpopulations (Fig 7E–7G), both *L. major* and *P. berghei* ANKA infections heightened the numbers of total splenic CD11b^+^ cells (Fig 7E). It is notable that *P. berghei* ANKA infection resulted in an increase in the number of inflammatory monocytes (CD11b^+^Ly6G^-^Ly6C^hi^) even in the presence of an ongoing *L. major* infection (Fig 7F). Conversely, an augmented presence of splenic neutrophils (CD11b^+^Ly6G^+^Ly6C^int^) was observed exclusively in mice infected with *L. major* only (Fig 7G). Additionally, it appears that the co-infection with *P. berghei* ANKA resulted in a reduction in the number of neutrophils recovered from the spleen (Fig 7G).

### *Plasmodium berghei* ANKA co-infection enhances systemic TNF levels in *L. major*-parasitized mice, compared to mice infected with *L. major* alone

*Plasmodium spp.* infection is known to induce substantial levels of systemic pro-inflammatory cytokines [39–42]. Pro-inflammatory cytokines, such as IFN-γ and TNF, play crucial roles in regulating CL [43]. To further investigate this interplay, we assessed serum levels of pro- and anti-inflammatory cytokines across our experimental groups.

**Fig 8:**
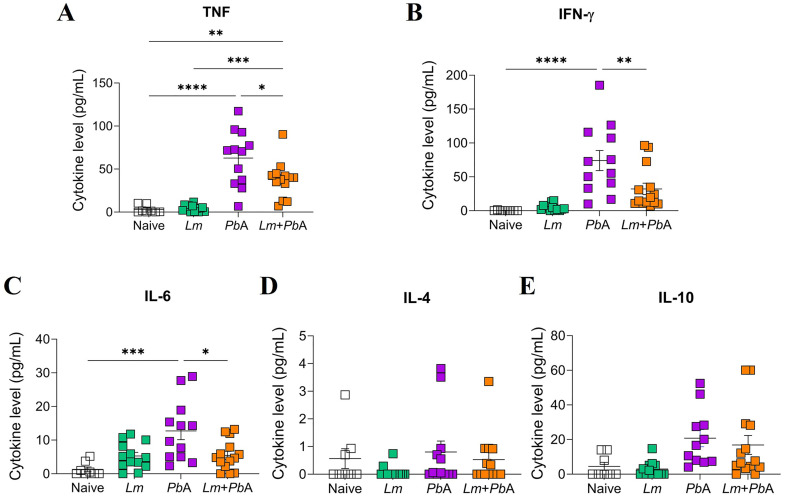
Modulation of serum cytokine levels following *P. berghei* ANKA co-infection in *L. major-*infected mice. Serum cytokine levels of the experimental groups on day 20 of *L. major* infection (corresponding to day 6 of *P. berghei* ANKA infection) were analyzed using the Cytometric Bead Array (CBA) Mouse Th1/Th2/Th17 kit. TNF (A), IFN-γ (B), IL-6 (C), IL-4 (D) and IL-10 (E). The experimental groups were designated as Naïve (uninfected animals), *Lm* (mice infected with *L. major* only), *Pb*A (mice infected with *P. berghei* ANKA only), and *Lm* + *Pb*A (mice co-infected with both *L. major* and *P. berghei* ANKA). The data represent a pool of three independent experiments, with n = 10-15 mice/group. Statistical analyses were performed by One-way ANOVA with Tukey’s multiple comparisons test. Values of p < 0.05 were considered statistically significant.

Mice infected solely with *P. berghei* ANKA exhibited elevated levels of TNF, IFN-γ, and IL-6 in comparison to uninfected mice (Fig 8A–8C). Interestingly, mice co-infected with both pathogens (*Lm* + *Pb*A group) exhibited reduced levels of these inflammatory cytokines (TNF, IFN-γ, and IL-6) compared to the *Pb*A group (Fig 8A–8C). However, it is worth noting that *P. berghei* ANKA co-infection resulted in a significant increase in TNF levels in mice with an ongoing *L. major* infection compared to mice infected with *L. major* alone (Fig 8A), which may contribute to improving *L. major* parasite control. The levels of other cytokines, including IL-4 (Fig 8D) and IL-10 (Fig 8E), demonstrated no statistically significant differences between the groups.

## Discussion

The co-infection of hosts with multiple parasites has been identified as a critical factor influencing the susceptibility and severity of numerous diseases. Simultaneous *Plasmodium* spp. infections, whether occurring naturally or in experimental settings, can disrupt the immune responses and affect the progression of other diseases, including those caused by viruses [44–46], bacteria [47,48], and parasites [49–51]. The clinical outcome of *Plasmodium* spp. - *Leishmania* spp. co-infection is influenced by a number of factors such as the parasite species, the order of infection, and host genetics. In natural infections, it is challenging to accurately measure and evaluate some of these variables. However, experimental models afford us the opportunity to elucidate the intricate details involved in co-infections with greater robustness and precision.

Infection of C57BL/6 mice with *L. major* (MHOM/IL/80/Friedlin) typically results in the development of a single lesion that heals spontaneously within a few weeks without the need for therapeutic intervention [23]. The present study demonstrated that co-infection with *P. berghei* ANKA in *L. major*-parasitized mice accelerated the healing process, significantly reducing lesion size and parasite load in the dermis just a few days following co-infection, in comparison to animals only infected with *L. major*. In a different murine experimental model, Pinna *et al*. (2016) demonstrated a comparable outcome, whereby the lesion and ulcerations were diminished in BALB/c animals co-infected with *L. braziliensis* and *P. yoelii* 17XNL (non-lethal strain) [52]. Moreover, a reduction in the parasite burden was observed in the lymph nodes of co-infected animals when compared to those infected only with *L. braziliensis* [52]. It is noteworthy that in another co-infection model, infection of C57BL/6 mice with *T. brucei brucei* was demonstrated to regulate the pathology of CL by *L. major*, resulting in a reduction of the lesion size and parasite load, the same parameters evaluated in our study [53]. Our findings align with these studies involving *P. yoelii* 17XNL and *T. brucei brucei*, indicating that the presence of a second parasite simultaneously in the same host can influence the clinical course of CL. However, studies employing *L. amazonensis* infection of BALB/c and C57BL/6 mice, followed by co-infection with *P. yoelii* 17XNL a few days later, demonstrated a worsening of skin lesions [52,54–56]. In this experimental context of *L. amazonensis* infection, concomitant plasmodial infection has the potential to exacerbate the severity of CL.

In addition, our study suggests that a previous infection with *L. major* did not prevent the development of eCM in animals co-infected with *P. berghei* ANKA. Moreover, no differences were observed in parasitemia, body weight, or temperature between the *P. berghei* ANKA-infected and co-infected animals. However, more subtle effects of co-infection with *L. major* on brain pathology cannot be excluded and warrant further investigation. The findings in the literature regarding the clinical course of a plasmodial infection in co-infected animals exhibit considerable variability, depending on the *Leishmania* spp. utilized in the study, as well as the mouse model employed. In brief, BALB/c mice co-infected with *L. braziliensis* and *P. yoelii* 17XNL exhibited a reduction in plasmodial parasitemia compared to mice infected only with *P. yoelii* 17XNL [52]. In contrast, BALB/c and C57BL/6 mice infected with *L. amazonensis* followed by *P. yoelii* 17XNL a few days later demonstrated a notable increase in parasitemia compared to those mice that had only been infected with *P. yoelii* 17XNL [52,54–56].

In infections caused by *Plasmodium* spp. and *Leishmania* spp., cell recruitment is a critical process that supports an effective or harmful immune response, which directly influences the clinical outcome of these diseases [57,58]. The analysis of the animals’ ears revealed that infection with *L. major* resulted in the expected recruitment of lymphoid and myeloid cells to the site of infection, as would be anticipated. However, in animals co-infected with both parasites, this recruitment was found to be reduced for both activated T cells, including IFN-γ^+^ CD4 T cells, and inflammatory monocytes. Notably, although we observed an increase in the percentage of IFN-γ ⁺ CD8 T cells in co-infected animals, this did not translate into an increase in their absolute number. This discrepancy probably reflects the overall decrease in total CD8 T cells in the tissue and suggests that the higher frequency does not represent a biologically significant increase in effector CD8 T cells. In the initial stages of *Leishmania* infection of the skin, IFN-γ plays a pivotal role in recruiting permissive monocytes, which are essential for the early establishment and proliferation of the parasite [59]. This finding suggests that co-infection with *P. berghei* ANKA limits cell migration to the site, thereby contributing to the reduction of the inflammatory process in the dermis.

The findings of this study are consistent with those of other experimental models, which have demonstrated the impact of co-infection with *Plasmodium* spp. on the recruitment of immune cells and the modulation of inflammation [51,60,61]. Teo *et al*. (2018) demonstrated that animals co-infected with chikungunya virus (CHIKV) and *P. berghei* ANKA exhibited a reduction in the infiltration of various immune cells in the joints, including CD4 T cells and monocytes, in comparison to those infected with the chikungunya virus alone [60]. This reduction in CD4 T cells was correlated with a decrease in joint inflammation, as the pathology is linked to IFN-γ-producing CD4 T cells [60]. Similarly, Vieira-Santos *et al*. (2021) observed that mice co-infected with *Ascaris suum* and *P. berghei* NK65-NY exhibited decreased cellularity in bronchoalveolar lavage, including reductions in lymphocytes, macrophages, and neutrophils [51]. In a separate model, Edwards *et al*. (2015) reported that co-infection of *P. chabaudi chabaudi* with murine pneumovirus significantly suppressed the recruitment of lymphocytes, neutrophils, and eosinophils to the lungs, in contrast to the recruitment observed in animals infected with the virus alone [61]. These studies indicate that co-infection with *Plasmodium* spp. plays a significant role in modulating the recruitment of different cell populations, which may contribute to the control of the inflammatory process or, in some cases, compromise the effectiveness of the immune response.

The restricted recruitment of cells to the site of infection can be attributed, at least in part, to mechanisms originating in secondary lymphoid organs, such as the lymph nodes and spleen. The evaluation of T cell populations in the lesion’s draining lymph node of co-infected mice also revealed a reduction in the number of T cells compared to those infected with *L. major* only. The reason for these decreases was not assessed in the present study; however, it may be associated with T cell apoptosis, as has been reported in the CHIKV + *P. berghei* ANKA and *P. yoelii* 17XNL + *Listeria monocytogenes* co-infection [60,62]. Moreover, co-infection can impact the migration of these cells. In the CHIKV + *P. berghei* ANKA model, co-infection not only induces apoptosis of T cells in lymph nodes but also suppresses the production of chemoattractant factors such as MIP-1α and MIP-1β, thereby limiting the migration of CD4 T cells to joints [60]. In a recent study, Foo *et al*. (2024) demonstrated that during co-infection with the neurotropic arbovirus SFV (Semliki Forest virus) and a non-neurotropic strain of influenza virus (IAV), there was an increase in influenza-specific CD8 T lymphocyte traffic to the brain, accompanied by augmented blood-brain barrier permeability [63]. Another potential mechanism for the observed reduction in T cells is the process of cell exhaustion, which has been demonstrated to impair the proliferation of these cells. Plasmodial infection has been shown to induce the expression of exhaustion molecules, including PD-1 and CTLA-4, in T cells [64–66]. In advanced stages of exhaustion, cells may enter apoptosis, thereby generating a state of immune hyporesponsiveness. This phenomenon has been described not only in *Plasmodium* spp. infections, but also in *Leishmania* spp. infections [67,68]. It is possible that co-infection may exacerbate this process.

Splenomegaly is a commonly observed phenomenon in the scientific literature concerning the infection of *Plasmodium* spp. [69–71]. The enlargement of the organ is attributed to the accumulation of RBCs and pRBCs, along with cell recruitment and clonal expansion [32]. This cellular expansion comprises the activation of T and B cells, which are essential for the control of the parasite [72,73]. The results of our study confirm that infection with *P. berghei* ANKA results in the expansion and activation of splenic CD4 and CD8 T cells. Although the overall numbers of these cells in animals co-infected with both parasites were lower than in those infected only with *P. berghei* ANKA, they were higher than in animals infected only with *L. major.* The data indicate that a prior infection with *L. major* had a partial inhibitory effect on the expansion of T cells following co-infection with *P. berghei* ANKA.

A type 1 immune response is strongly induced in C57BL/6 mice during malaria and leishmaniasis, resulting in the production of pro-inflammatory cytokines such as IFN-γ and TNF [57,74,75]. Nevertheless, research has demonstrated that co-infections involving *Plasmodium* spp., whether with viral, bacterial, or parasitic pathogens, result in a distinct alteration of the serum cytokine profile in comparison to single-pathogen infections [52,76,77]. Our findings align with these observations, as evidenced by the data, which indicate that co-infection with *P. berghei* ANKA in previously *L. major*-infected animals does not result in an elevation of type 1 cytokines (IFN-γ and IL-6) observed in animals infected with *P. berghei* ANKA alone. However, animals co-infected with *L. major* and *P. berghei* ANKA exhibited elevated serum TNF levels in comparison to those infected with *L. major* alone. This may contribute to the control of *L. major* parasite load in the lesion.

In brief, our study elucidated the immunological and clinical consequences of *Plasmodium* spp. – *Leishmania* spp. co-infection demonstrating that infection with *P. berghei* ANKA modulates the progression of ongoing *L. major* infection by a reduction in lesion size and parasite burden in the dermis, and reduction of activated T cells and inflammatory monocytes recruitment to the site of infection. Although co-infection with *P. berghei* ANKA resulted in an increase in the pro-inflammatory cytokine TNF that may be related to the control of the *L. major* parasite load, co-infection exerted an immunomodulatory effect that attenuated local inflammation, minimizing tissue damage and accelerating the healing process. However, the precise mechanisms underlying this cross-regulation, including the signaling pathways and molecular interactions, remain to be fully elucidated.

## Supporting information

S1 Fig Experimental design. On day 0, the *Lm* and the *Lm* + *Pb*A groups received 2 x10^5^ metacyclic forms of *L. major* intradermally (ID) in the ears. Subsequently, on either the 14^th^ or 28^th^ day, depending on the experiment and detailed in the legends of the subsequent figures, the *Lm* + *Pb*A and *Pb*A groups received an intraperitoneal inoculation (IP) of 10^6^ pRBCs infected with *P. berghei* ANKA. The uninfected animals (Naive group) received intradermal and intraperitoneal inoculations of RPMI and PBS, respectively, at the corresponding time points. On the 20^th^ or 34^th^ day following *L. major* infection (which corresponds to a period of 6 days after *P. berghei* ANKA infection of the *Pb*A and *Lm* + *Pb*A groups), all groups were euthanized for analyses. In some experiments, the size of the lesion and the survival rate were monitored over time. Mouse illustration obtained from Openclipart (https://openclipart.org), public domain (CC0 license).
(TIF)

S2 Fig Representative plots illustrating the gating strategy applied to identify the T cells and myeloid cells in the ear. **(A)** After gating on “all cells” (i), singlets (ii), and CD3^+^ cells (iii), the CD4 and CD8 T cells were defined (iv). Activated CD4 (CD3^+^CD4^+^CD62L^-^) (v) and CD8 (CD3^+^CD8^+^CD62L^-^) (vi) T cells were defined based on the lack of CD62L expression, and among them, the T cells expressing IFN-γ were further characterized (vii and viii). Identification of positive populations was facilitated by employing the fluorescence minus one (FMO) control as a negative reference. **(B)** After gating on “all cells” (i), singlets (ii), and CD11b^+^ cells (iii), the neutrophils (CD11b^+^Ly6G^+^Ly6C^int^) and monocytes (CD11b^+^Ly6G^-^Ly6C^hi^) were defined.
(TIF)

S3 Fig Representative plots illustrating the gating strategy applied to identify the T cells in the draining lymph node. After gating on “all cells” (i), singlets (ii), and CD3^+^ cells (iii), the CD4 and CD8 T cells were defined (iv). Activated CD4 (CD3^+^CD4^+^CD62L^-^) (v) and CD8 (CD3^+^CD8^+^CD62L^-^) (vi) T cells were defined based on the lack of CD62L expression. Identification of positive populations was facilitated by employing the fluorescence minus one (FMO) control as a negative reference.
(TIF)

S4 Fig Representative plots illustrating the gating strategy applied to identify the T cells and myeloid cells in the spleen. **(A)** After gating on “all cells” (i), singlets (ii), and CD3^+^ cells (iii), the CD4 and CD8 T cells were defined (iv). Activated CD4 (CD3^+^CD4^+^CD62L^-^) (v) and CD8 (CD3^+^CD8^+^CD62L^-^) (vi) T cells were defined based on the lack of CD62L expression. Identification of positive populations was facilitated by employing the fluorescence minus one (FMO) control as a negative reference. **(B)** After gating on “all cells” (i), singlets (ii), and CD11b^+^ cells (iii), the neutrophils (CD11b^+^Ly6G^+^Ly6C^int^) and monocytes (CD11b^+^Ly6G^-^Ly6C^hi^) were defined.
(TIF)

S5 Fig Total number of IFN-γ^+^ T cells is not altered during coinfection. Flow cytometry was employed to assess the total number of IFN-γ^+^ CD4 T cells (A) and IFN-γ^+^ CD8 T cells (B) present in ear of the experimental groups on day 20 of *L. major* infection (corresponding to day 6 of *P. berghei* ANKA infection). The experimental groups were designated as Naïve (uninfected animals), *Lm* (mice infected with *L. major* only), and *Lm* + *Pb*A (mice co-infected with both *L. major* and *P. berghei* ANKA). The data are representative of two independent experiments with n = 4–8 mice/group. Statistical analyses were performed by One-way ANOVA with Tukey’s multiple comparisons test. Values of p < 0.05 were considered statistically significant.
(TIF)
