# Supplementary figures and images for: Impact of co-infection with Plasmodium berghei ANKA in Leishmania major-parasitized mice on immune modulation and cutaneous leishmaniasis
Source: PLoS Negl Trop Dis. 2025 Jul 28;19(7):e0013302. doi: 10.1371/journal.pntd.0013302 (PMC12316397; doi:10.1371/journal.pntd.0013302)

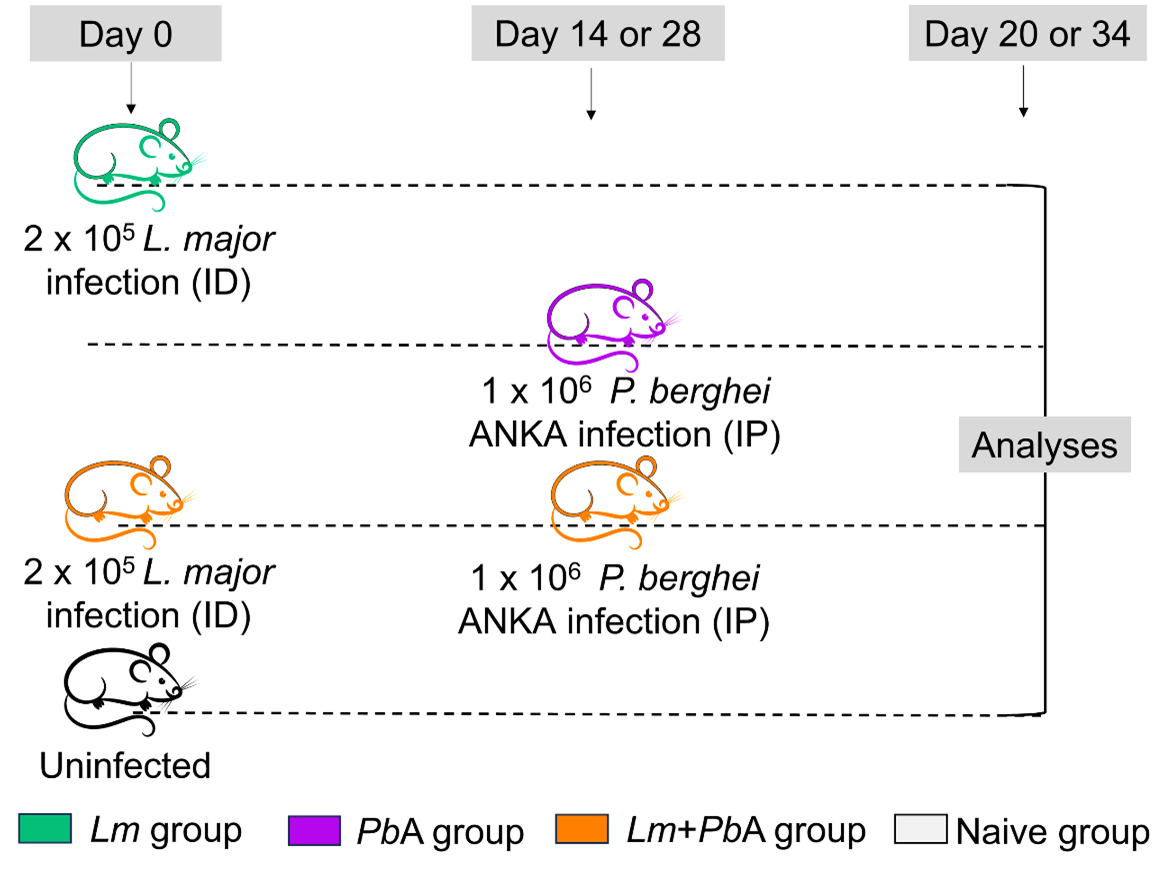

Supplement: S1 Fig — On day 0, the Lm and the Lm + PbA groups received 2 x10^5^ metacyclic forms of L. major intradermally (ID) in the ears. Subsequently, on either the 14th or 28th day, depending on the experiment and detailed in the legends of the subsequent figures, the Lm + PbA and PbA groups received an intraperitoneal inoculation (IP) of 106 pRBCs infected with P. berghei ANKA. The uninfected animals (Naive group) received intradermal and intraperitoneal inoculations of RPMI and PBS, respectively, at the corresponding time points. On the 20th or 34th day following L. major infection (which corresponds to a period of 6 days after P. berghei ANKA infection of the PbA and Lm + PbA groups), all groups were euthanized for analyses. In some experiments, the size of the lesion and the survival rate were monitored over time. Mouse illustration obtained from Openclipart (https://openclipart.org), public domain (CC0 license). (TIF) [file pntd.0013302.s001.tif]

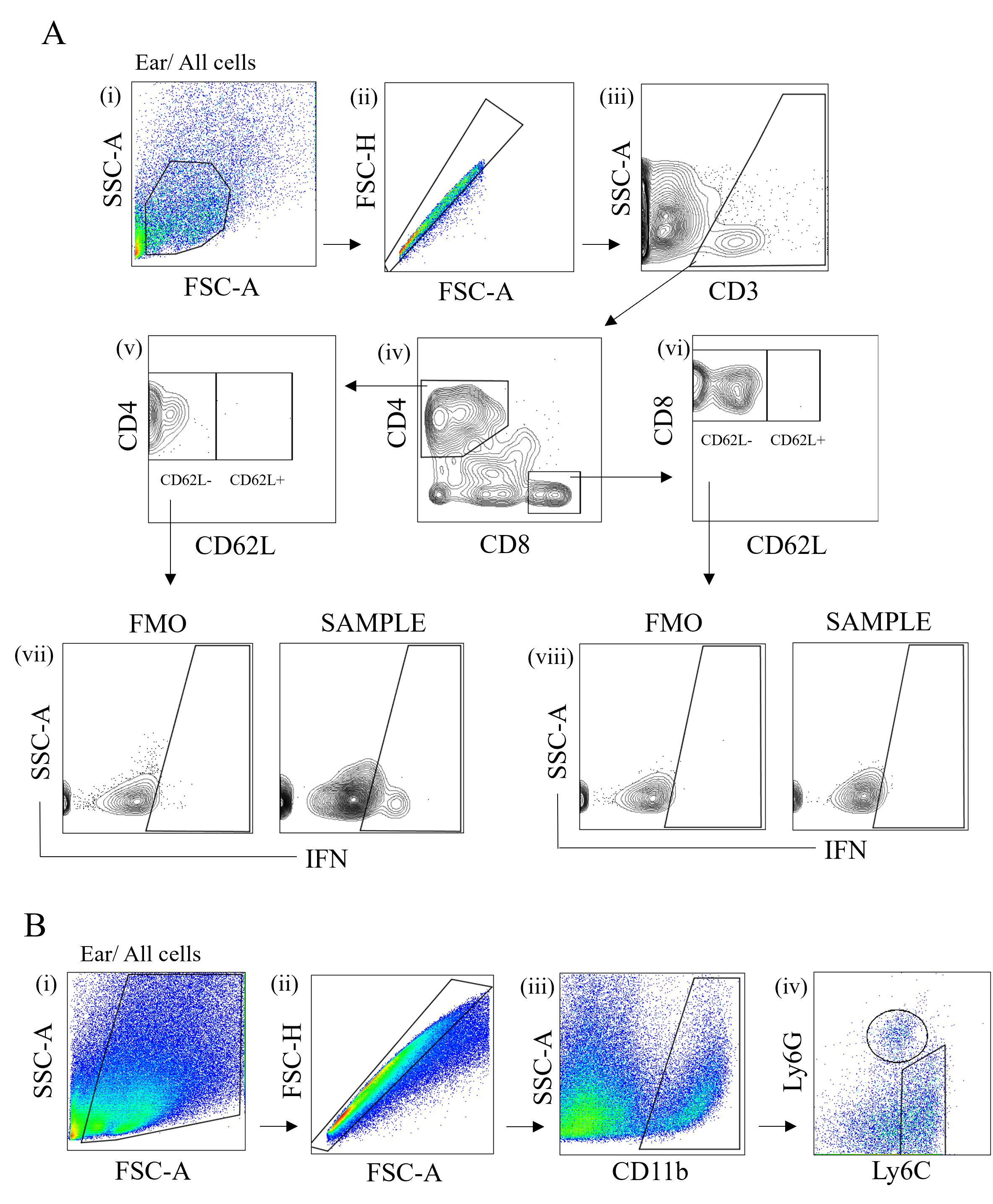

Supplement: S2 Fig — (A) After gating on “all cells” (i), singlets (ii), and CD3+ cells (iii), the CD4 and CD8 T cells were defined (iv). Activated CD4 (CD3+CD4+CD62L-) (v) and CD8 (CD3+CD8+CD62L-) (vi) T cells were defined based on the lack of CD62L expression, and among them, the T cells expressing IFN-γ were further characterized (vii and viii). Identification of positive populations was facilitated by employing the fluorescence minus one (FMO) control as a negative reference. (B) After gating on “all cells” (i), singlets (ii), and CD11b+ cells (iii), the neutrophils (CD11b+Ly6G+Ly6Cint) and monocytes (CD11b+Ly6G-Ly6Chi) were defined. (TIF) [file pntd.0013302.s002.tif]

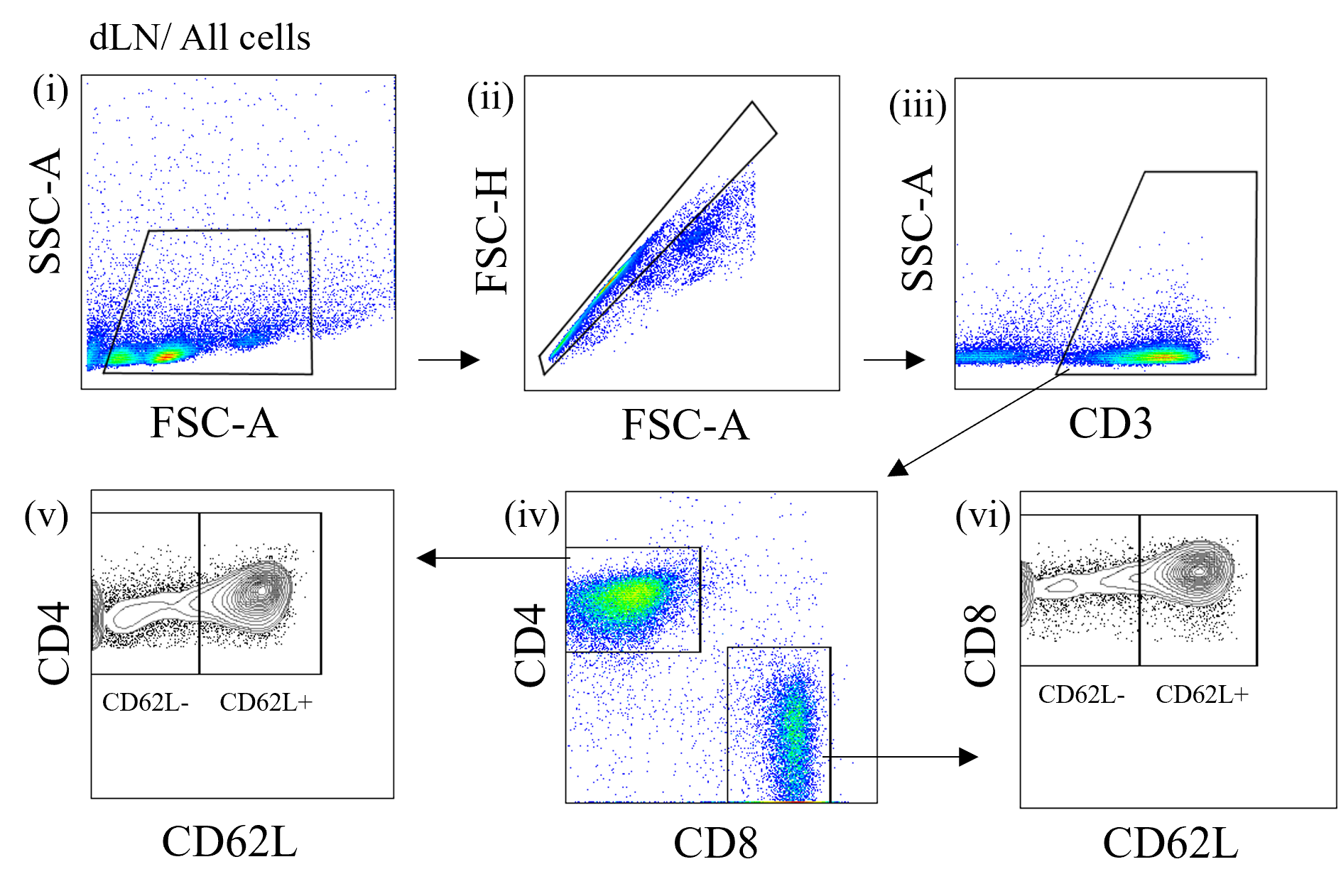

Supplement: S3 Fig — After gating on “all cells” (i), singlets (ii), and CD3+ cells (iii), the CD4 and CD8 T cells were defined (iv). Activated CD4 (CD3+CD4+CD62L-) (v) and CD8 (CD3+CD8+CD62L-) (vi) T cells were defined based on the lack of CD62L expression. Identification of positive populations was facilitated by employing the fluorescence minus one (FMO) control as a negative reference. (TIF) [file pntd.0013302.s003.tif]

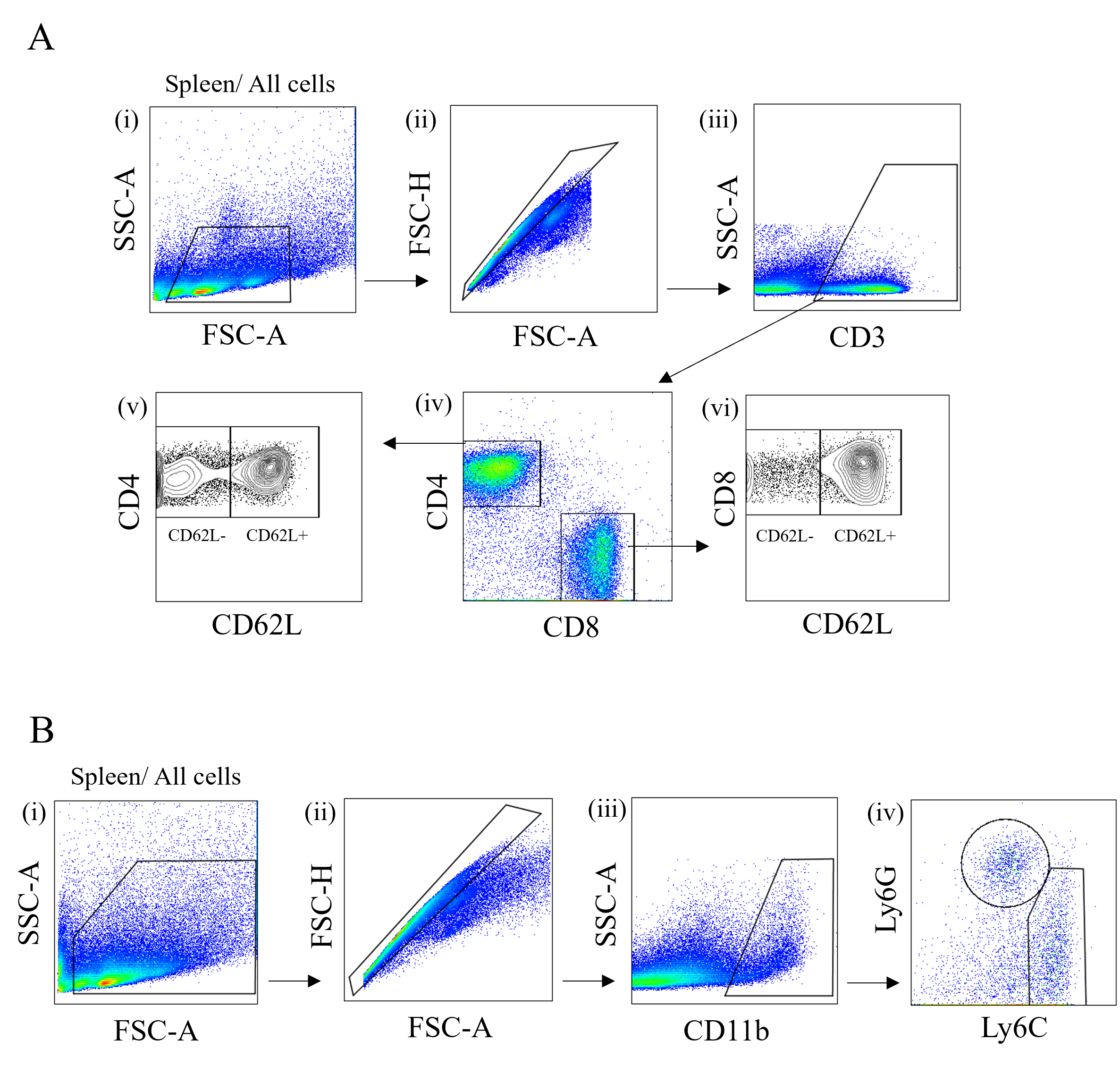

Supplement: S4 Fig — (A) After gating on “all cells” (i), singlets (ii), and CD3+ cells (iii), the CD4 and CD8 T cells were defined (iv). Activated CD4 (CD3+CD4+CD62L-) (v) and CD8 (CD3+CD8+CD62L-) (vi) T cells were defined based on the lack of CD62L expression. Identification of positive populations was facilitated by employing the fluorescence minus one (FMO) control as a negative reference. (B) After gating on “all cells” (i), singlets (ii), and CD11b+ cells (iii), the neutrophils (CD11b+Ly6G+Ly6Cint) and monocytes (CD11b+Ly6G-Ly6Chi) were defined. (TIF) [file pntd.0013302.s004.tif]

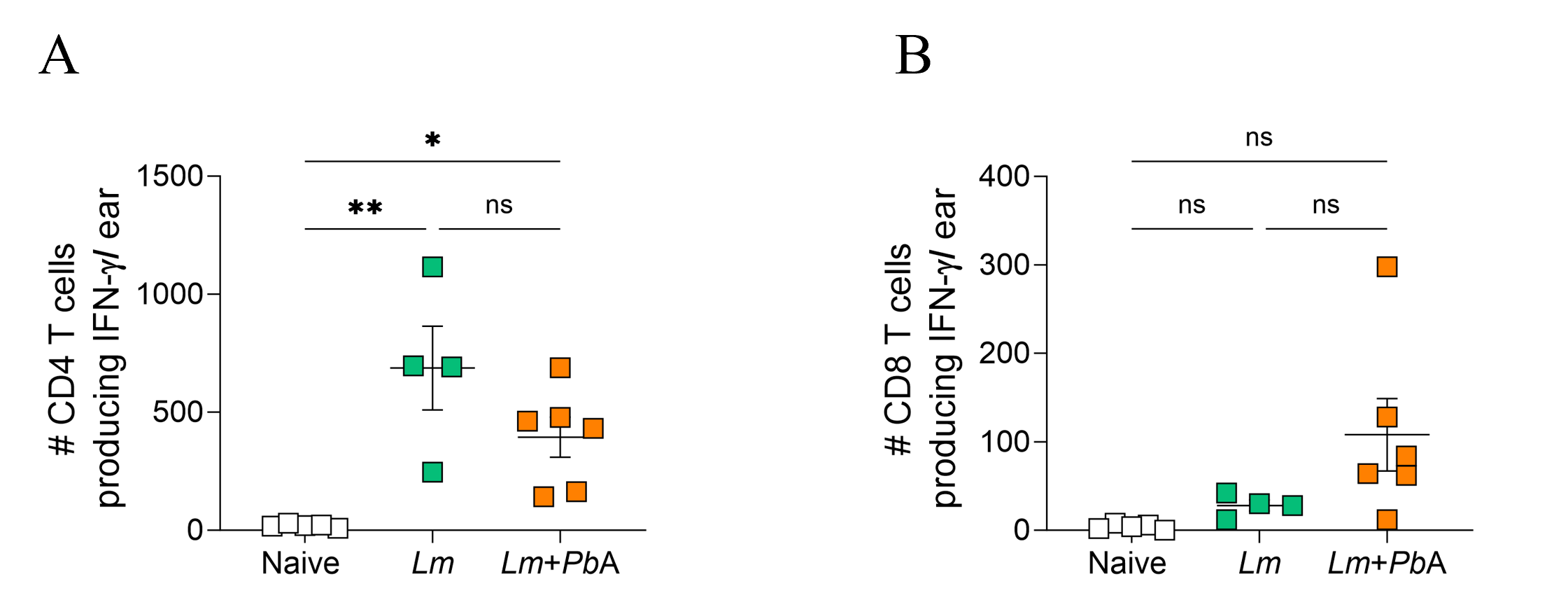

Supplement: S5 Fig — Flow cytometry was employed to assess the total number of IFN-γ+ CD4 T cells (A) and IFN-γ+ CD8 T cells (B) present in ear of the experimental groups on day 20 of L. major infection (corresponding to day 6 of P. berghei ANKA infection). The experimental groups were designated as Naïve (uninfected animals), Lm (mice infected with L. major only), and Lm + PbA (mice co-infected with both L. major and P. berghei ANKA). The data are representative of two independent experiments with n = 4–8 mice/group. Statistical analyses were performed by One-way ANOVA with Tukey’s multiple comparisons test. Values of p < 0.05 were considered statistically significant. (TIF) [file pntd.0013302.s005.tif]
